# Evaluation of Antimalarial Potential of Aqueous Crude *Gymnema Inodorum* Leaf Extract against *Plasmodium berghei* Infection in Mice

**DOI:** 10.1155/2021/9932891

**Published:** 2021-04-27

**Authors:** Sakaewan Ounjaijean, Chonticha Romyasamit, Voravuth Somsak

**Affiliations:** ^1^Research Institute for Health Sciences, Chiang Mai University, Chiang Mai 50200, Thailand; ^2^School of Allied Health Sciences, Walailak University, Nakhon Si Thammarat 80160, Thailand; ^3^Research Excellence Center for Innovation and Health Products, Walailak University, Nakhon Si Thammarat 80160, Thailand

## Abstract

Malaria is still a serious cause of mortality and morbidity. Moreover, the emergence of malaria parasite resistance to antimalarial drugs has prompted the search for new, effective, and safe antimalarial agents. For this reason, the study of medicinal plants in discovering new antimalarial drugs is important and remains a crucial step in the fight against malaria. Hence, this study is aimed at investigating the antimalarial activity of *Gymnema inodorum* leaf extract (GIE) in *Plasmodium berghei* infected mice. Aqueous crude extract of *G. inodorum* leaves was prepared in distilled water (DW) and acute toxicity in mice was carried out. The antimalarial activity was assessed in the five groups of ICR mice employing the 4-day suppressive and curative tests. Untreated and positive controls were given DW along with 10 mg/kg of chloroquine, respectively. Any signs of toxicity, behavioral changes, and mortality were not observed in mice given GIE up to 5,000 mg/kg. GIE significantly (*P* < 0.05) suppressed parasitemia by 25.65%, 38.12%, and 58.28% at 10, 50, and 100 mg/kg, respectively, in the 4-day suppressive test. In the curative test, the highest parasitemia inhibition of 66.78% was observed at 100 mg/kg of GIE. Moreover, GIE prevented packed cell volume reduction and body weight loss compared to the untreated control. Additionally, GIE was able to prolong the mean survival time of infected mice significantly. The results obtained in this study confirmed the safety and promise of *G. inodorum* as an important source of new antimalarial agents and justify its folkloric use for malaria treatment.

## 1. Introduction

Though malaria mortality rates have reduced steadily over the period 2000–2018, about 229 million cases and 409,000 deaths in 2019 showing that malaria is still a serious infectious disease with 95% of the cases and deaths occurring in Africa, especially in children under five years of age [1]. Malaria is caused by *Plasmodium* parasites transmitted by the female *Anopheles* mosquito. Although an effective vaccine is the best control for malaria, there is no commercially available malarial vaccine, and it is still ongoing currently [2]. The strategy for malaria and case management have largely relied and mainly focus on antimalarial drugs [3]. However, the emergence of *Plasmodium* parasite resistance to currently available antimalarial drugs such as amodiaquine, mefloquine, quinine, chloroquine, sulfadoxine-pyrimethamine, and artemisinin derivatives has been reported [4]. Therefore, searching for new and novel antimalarial compounds that are safe, effective, and affordable are urgently needed. The great significance of plant-derived antimalarial drugs is highlighted by quinine (derived from Cinchona tree) and artemisinin (derived from *Artemisia annua*). In this respect, plant resources play a crucial role as a potential target for new antimalarial drugs. World Health Organization estimated that about 80% of malaria endemic countries rely on traditional medicines to treat the disease. However, scientific reports to validate the antimalarial properties of these traditional remedies are not yet fully explored [5].

*Gymnema sylvestre*, belonging to family Asclepiadaceae, is found in tropical and subtropical regions in central and southern India, southern part of China, Africa, Malaysia, and Sri Lanka [6]. It has been one of the major botanicals used in Ayurvedic medicine to treat disease conditions ranging from diabetes, malaria, to snakebites [7]. *In vitro* and *in vivo* investigation *G. sylvestre* revealed the pharmacological potentials including antidiabetic and hypoglycemic, antihyperlipidemic, antioxidant, anti-inflammatory, antiarthritic, anticancer, antimicrobial, hepatoprotective, cardioprotective, and immunosuppressive activities [8]. These actions are attributed to the therapeutically essential families of compounds, including polyphenols, flavonoids, kaempferol, quinones, anthraquinones, tannins, triterpenoid saponins, and gymnemic acids [9]. *Gymnema inodorum* (Lour.) Decne (Thai name: Phak Chiang Da), belonging to the same group as *G. sylvestre*, is found mostly in South-Eastern Asia, including Thailand, especially in the Northern and Eastern parts of the country [10]. *G. inodorum* extract has been shown to have an ability to inhibit glucose absorption as well as potent antioxidant activity with polyphenols, flavonoids, and gymnemic acids as the major compounds [11, 12]. The plant is endowed with active compounds such as polyphenols, flavonoids, terpenoids, and saponin, with reported antioxidant and antimalarial activities [5]. Therefore, this also justified the present study. Considering all the above since no previous study for antimalarial effect of *G. inodorum* is found essential, hence, this study was designed to evaluate *G. inodorum* leaf extract's antimalarial activity against *Plasmodium berghei* infection in mice.

## 2. Materials and Methods

### 2.1. Chemicals and Reagents

Chemicals and chloroquine diphosphate salt (CQ) were purchased from Sigma (Sigma-Aldrich, St. Louis, MO, USA). All reagents and solvents used were of analytical grade. CQ was prepared in distilled water (DW) at 10 mg/kg and kept at 4°C until used.

### 2.2. Plant Material and Preparation of Extract

Leaves of *Gymnema inodorum* were collected from the Chiangda Organic Company Garden, Chiang Mai, Thailand. A plant biologist authenticated this plant at Chiang Mai University and it was subsequently deposited at the Research Excellence Center for Innovation and Health Products, Walailak University. The plant materials were dried at 50°C in a hot-air oven, and the electric blender was then used to obtained dried powdered material. For the preparation of the extract, 250 g of the dried powdered *G. inodorum* was soaked in 750 ml of DW 80°C for 20 min occasional stirring. After filtration with Whatman no. 1 filter paper, the filtrate was concentrated using lyophilization. The dried powdered form of aqueous crude extract of *G. inodorum* (GIE) was stored at −20°C until further use [11].

### 2.3. Mice

The present study was conducted with a total of 40 healthy and nonpregnant male mice. ICR mice aged 4–5 weeks old, weighing 20–25 g, obtained from the Nomura Siam International Co., Ltd., were used. They were acclimatized for at least one week before being used for the experiments in the animal house at 25°C with 12 h light-12 h dark cycle, with the standard pellet diet and drinking water, *ad libitum*. According to the National Institute of Health Guideline for Care and Use of Laboratory Animals, the experiments involving animals were conducted. The protocols have been ratified and approved by the appropriate animal ethics committee, Walailak University (WU-AICUC-63-031).

### 2.4. Acute Toxicity Assay

The acute toxicity assay of GIE was determined by the modified Lorke's method as described previously [13]. Naïve ICR mice were randomly divided into four groups (3 mice per group) and starved for 3 h before the experiment began. The GIE at doses of 1,000, 3,000, and 5,000 mg/kg was administered orally by gavage. The control group was given 10 ml/kg of DW. Then, the mice were observed for any manifestation of toxicity, including paw licking, stretching of the entire body, salivation, weakness, respiratory distress, and death in the first 4 h and after that daily for 14 days.

### 2.5. Rodent Malaria Parasite

Chloroquine-sensitive *Plasmodium berghei* strain ANKA (PbANKA) was obtained from Malaria Research and Reference Reagent Resource Center (MR4) and used for the experiments. A cryopreserved parasite stock was thawed in a water bath at 37°C and then inoculated intraperitoneally into naïve recipient ICR mice. Parasitemia was monitored daily in the tail vein by microscopic examination of Giemsa-stained thin blood smear. Subpassage of intraperitoneal infection in new mice consisting of 1 × 10^7^ infected erythrocytes in a total volume of 0.2 ml was done on a weekly basis.

### 2.6. Measurement of Parasitemia

Mouse blood was collected from the tail snip of each mouse, and a thin blood smear was then prepared on microscopic slides. The smeared slides were fixed with absolute methanol and stained with 10% Giemsa solution for 10 min at room temperature. The stained slides were subsequently gently rinsed with tap water and dried at room temperature. The infected erythrocytes were counted using a light microscope under oil immersion of 100x magnification. Percent parasitemia was calculated using the following formula:

(1) $$\begin{array}{c} \%\text{ parasitemia}=\frac{\text{number of infected erythrocytes}\times 100}{\text{total number of erythrocytes}}. \end{array}$$

### 2.7. Measurement of Packed Cell Volume

Mouse blood was collected from the tail vein of each mouse and filled to 3/4^th^ in heparinized capillary tubes. The tubes were sealed at one end with a sealing cray and then centrifuged at 12,000 rpm for 5 min using a microhematocrit centrifuge. Packed cell volume (PCV) was determined using a standard hematocrit reader.

### 2.8. Measurement of Body Weight

The body weight (BW) of each mouse in all experimental groups was measured using a sensitive digital weighing balance on the day before infection and after experimental treatment.

### 2.9. Chemosuppressive Antimalarial Test

Chemosuppressive antimalarial assay was carried out with standard 4-day suppressive test as previously described [14]. Groups of naïve ICR mice (3 mice of each) were inoculated with 1 × 10^7^ PbANKA infected erythrocytes by intraperitoneal injection. These infected mice were then randomly divided into five groups. The GIE at the doses of 10, 50, and 100 mg/kg was administered orally by gavage once a day for 4 consecutive days (day 0–day 3). The untreated and positive control groups were given 10 ml/kg of DW and 10 mg/kg of CQ, respectively. Additionally, normal ICR mice were also used as healthy control. On day 4, PCV was measured, and parasitemia was also estimated. The percentage of inhibition was subsequently calculated using the following formula:

(2) $$\begin{array}{c} \text{\% inhibition}=\frac{\left(\text{parasitemia of untreated group}-\text{parasitemia of tested group}\right)}{\text{parasitemia of untreated group}}\times 100\text{.} \end{array}$$

### 2.10. Curative Antimalarial Test

Evaluation of the curative potential of GIE was carried out according to the method of Rane's test as described previously [15]. Naïve ICR mice were inoculated intraperitoneally with 1 × 10^7^ PbANKA infected erythrocytes. Seventy-two hours after infection, these infected mice were randomly divided into five groups of 3 mice each. They were administered orally by gavage with 10, 50, and 100 mg/kg of GIE, 10 mg/kg of CQ as a positive control, and 10 ml/kg of DW as an untreated control. The treatment was carried out once a day for 4 consecutive days, and blood was then collected to measure PCV and parasitemia. The percentage of inhibition was calculated according to the formula indicated above. In addition, normal ICR mice were also used as healthy control.

### 2.11. Measurement of Mean Survival Time

In all in vivo antimalarial experiments, mortality was monitored, and the number of days from the time of infection up to death was then recorded for each mouse for 30 days. The mean survival time (MST) was calculated according to the following formula:

(3) $$\begin{array}{c} \text{MST}=\frac{\text{sum of survival time of all mice in a group}}{\text{total number of mice in the group}}. \end{array}$$

### 2.12. Statistical Analysis

All results were presented as mean ± standard error of mean (SEM) and analyzed by one-way ANOVA followed by a post-test (Tukey-Kramer multiple comparison tests) using the GraphPad Prism version 6 (GraphPad Prism Software, San Diego, CA). Statistical significance was considered at 95% confidence, *P* < 0.05.

## 3. Results

### 3.1. Acute Toxicity Assay

The acute toxicity assay indicated that GIE did not cause mortality of ICR mice within 24 h as well as in the next 14 days up to 5,000 mg/kg. Gross physical and behavioral observation of the treated mice also presented no visible signs of toxicity. Moreover, they were physically active after GIE administration for over 14 days. LD50 of the GIE was greater than 5,000 mg/kg.

### 3.2. Propagation of Plasmodium berghei Infected Mice

**Figure 1:**
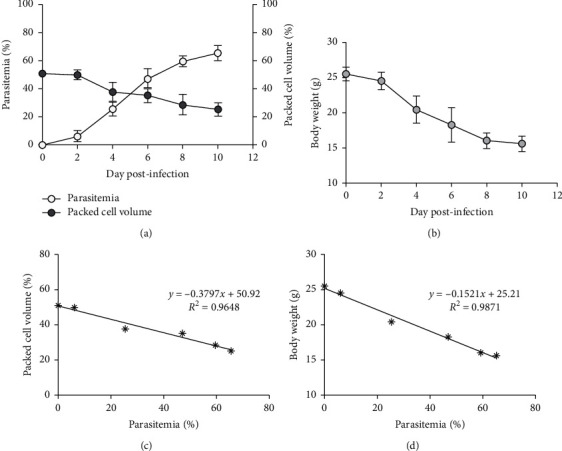
PbANKA infection. (a) Parasitemia, PCV, and (b) BW of ICR mice infected with 1 × 10^7^ PbANKA infected erythrocytes were monitored on different days after infection. The correlation was also assessed between (c) parasitemia and PCV, and (d) parasitemia and BW. Results were presented as mean ± SEM.

PbANKA propagation in mice was shown by increasing parasitemia as presented in Figure 1(a). Parasitemia was first detectable on day one after infection and reached 65% on day 10. Next, we observed that PCV was markedly decreased in PbANKA infected mice. BW loss during PbANKA infection was also observed (Figure 1(b)). Additionally, we also observed that parasitemia was negatively correlated with PCV (*R*^2^ = 0.9648) and BW (*R*^2^ = 0.9871), as presented in Figures 1(c) and 1(d), respectively.

### 3.3. Chemosuppressive Antimalarial Test

**Figure 2:**
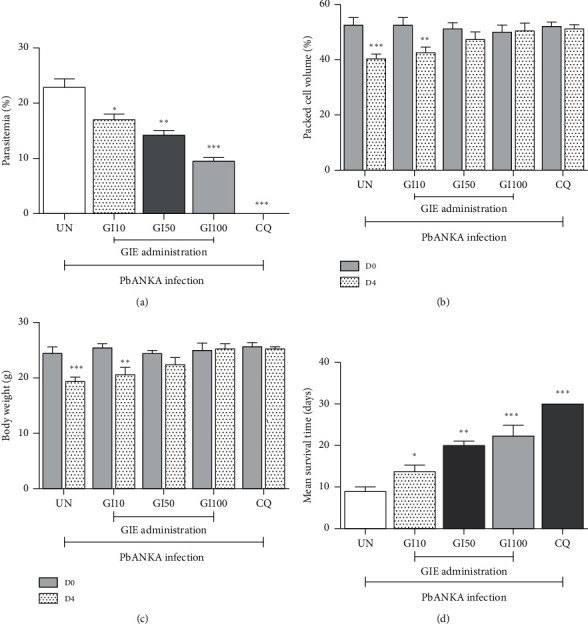
Chemosuppressive antimalarial activity of GIE against PbANKA infected mice. Groups of ICR mice were infected with 1 × 10^7^ PbANKA infected erythrocytes and administered orally with 10, 50, and 100 mg/kg of GIE for 4 consecutive days. (a) Parasitemia, (b) PCV, and (c) BW were measured. (d) MST was also determined. Results were presented as mean ± SEM. UN: untreated mice, GI10: 10 mg/kg of GIE treated mice, GI50: 50 mg/kg of GIE treated mice, GI100: 100 mg/kg of GIE treated mice, and CQ: 10 mg/kg of chloroquine treated mice.  ^*∗*^*P* < 0.05,  ^*∗∗*^*P* < 0.01, and  ^*∗∗∗*^*P* < 0.001 compared to either UN or D0 after infection.

Chemosuppressive antimalarial test of GIE in PbANKA infected mice resulted in dose-dependent reduction in parasitemia as compared to respective untreated control. The positive group treated with 10 mg/kg of CQ cleared the parasitemia on day 4. GIE at 10, 50, and 100 mg/kg showed 25.65%, 38.12%, and 58.28% inhibition, respectively, and the results were significant (*P* < 0.05), compared to the untreated control at day 4 (Figure 2(a)).

As indicated in Figures 2(b) and 2(c), PbANKA infection induced a significant (*P* < 0.001) decrease of PCV and BW in the untreated group. GIE at the doses of 50 and 100 mg/kg was able to prevent the PbANKA infected mice from PCV reduction and BW loss at day 4. No difference was observed between 50 and 100 mg/kg of GIE in protecting PCV and BW in the infected mice. However, 10 mg/kg of GIE did not protect the reduction of PCV and BW loss in the infected mice. Additionally, all three doses of GIE in the treated groups were correlated with significant (*P* < 0.05) increased MST, compared to the untreated control (Figure 2(d)).

### 3.4. Curative Antimalarial Test

**Figure 3:**
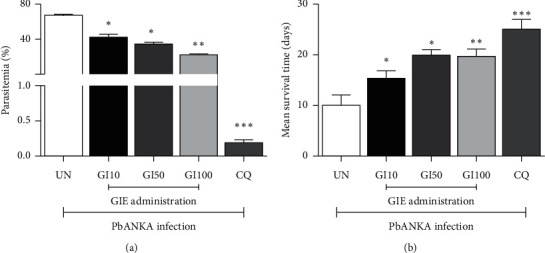
Curative antimalarial activity of GIE against PbANKA infected mice. Groups of ICR mice were infected with 1 × 10^7^ PbANKA infected erythrocytes for 72 h and subsequently treated orally with 10, 50, and 100 mg/kg of GIE for 4 consecutive days. (a) Parasitemia and (b) MST were determined. Results were presented as mean ± SEM. UN: untreated mice, GI10: 10 mg/kg of GIE treated mice, GI50: 50 mg/kg of GIE treated mice, GI100: 100 mg/kg of GIE treated mice, and CQ: 10 mg/kg of chloroquine treated mice.  ^*∗*^*P* < 0.05,  ^*∗∗*^*P* < 0.01, and  ^*∗∗∗*^*P* < 0.001 compared to UN.

It was observed that GIE produced a dose-dependent reduction in parasitemia in the extract-treated groups as compared to the untreated control (Figure 3(a)). The parasitemia inhibition of the extract-treated groups was 35.72%, 48.50, and 66.78% for the 10, 50, and 100 mg/kg of GIE, respectively, while that of the CQ treated group was 99.73%. Concerning the MST, the results showed that all three doses of GIE and CQ were able to significantly (*P* < 0.05) prolong MST as compared to the untreated control (Figure 3(b)).

## 4. Discussion

The antimalarial activity of *G. inodorum* leaf extract against *P. berghei* infected mice in chemosuppressive and curative models was reported. Oral administration of GIE did not cause mortality or any sign of behavioral changes until the end of 14 days up to a dose of 5,000 mg/kg. According to the Organization for Economic Cooperation and Development (OECD) guideline of acute toxicity assay, if LD_50_ of the GIE (>5,000 mg/kg) was found to be three times the minimum effective dose (50 and 100 mg/kg), it was taken as a good candidate for further studies [16]. This could justify the routine use of GIE in the traditional management of malaria and other ailments.

The antimalarial activities of the GIE were investigated using the standard models. The *in vivo* models using rodent malaria have been validated by identifying standard antimalarial drugs, including CQ, pyrimethamine, and artemisinin derivatives [14]. It is also cost-effective in conducting the pharmacological screening in rodent models than in primate models. Accordingly, the GIE showed dose-dependent inhibition of parasitemia with significant. A compound is considered active when the inhibition in parasitemia is ≥30%, which is in agreement and supports the finding of this study [17]. Hence, GIE can be considered to be active in its schizontocidal activity against PbANKA infected mice. The antimalarial activity of the crude plant extracts is due to bioactive compounds, such as polyphenols, flavonoids, alkaloids, terpenoids, and saponin. Therefore, the antimalarial activity of GIE could have resulted from the single or combined action of the above compounds [18]. The possible actions of antimalarial activity might be through the antioxidant, immunomodulatory, intercalation in DNA, blocking of protein synthesis, inhibition of erythrocyte invasion by parasites, disruption of hemozoin formation, or by other unknown mechanisms [3–5, 17]. However, the lower dose (10 mg/kg) of GIE may be considered to have lower antimalarial activity. This could be due to the presence of active compounds at low levels in the GIE.

The PCV reduction is one feature of both human and rodent malaria infection. PbANKA infected mice suffer from hemolysis and severe anemia because of rapid destruction and clearance of erythrocytes by increased parasitemia [19]. Moreover, PbANKA infection also increased erythrocyte fragility, erythropoietic suppression, and dyserythropoiesis [19]. Plant extracts with antimalarial activity are expected to prevent PCV reduction and hemolysis. It was noted that the effective prevention of PCV reduction in the GIE treated groups at the doses of 50 and 100 mg/kg might indicate the protective role of this extract. This might result from the antioxidant and antimalarial effects of the GIE against PbANKA infected erythrocytes. Loss of BW is another manifestation of rodent malaria infection in mice. This might be due to disturbed metabolic function, catabolic activity on lipids, anorexigenic effect, appetite depressive action, and malaria-associated hypoglycemia [20, 21]. Infected mice treated with 50 and 100 mg/kg of GIE showed the protection of BW loss; hence, this might tell us about the antimalarial activity of the extract. Moreover, appetite-stimulating activity and the function of vitamin B such as B1, B2, and B3 might also be considered [22, 23]. However, 10 mg/kg of GIE was not strong enough to significantly prevent PCV reduction and BW loss. It could be due to the low level of active compounds associated with the extract.

The antimalarial effect of GIE on an established infection of PbANKA in mice was carried out. GIE at all doses presented a significant dose-dependent curative effect. This might be an indication that GIE had effective antimalarial activity in the late stage of infection. The percentage of inhibition of the curative test was found to be high compared to the chemosuppressive test. This might be due to the stage-specific action of active compounds presented in GIE. On the other hand, MST is also an important parameter to evaluate the antimalarial activity of the extract [17]. A plant extract that can prolong MST of infected mice compared to untreated control is considered active. Our results showed that GIE at all doses, compared to untreated control, had significantly prolonged MST, which was directly linked to parasitemia inhibition in both tested models.

## 5. Conclusion

Acute toxicity assay conducted on GIE confirmed the safety of this plant up to 5,000 mg/kg. The present study's findings indicated that GIE exerted significant chemosuppressive and curative antimalarial activities against PbANKA. The highest antimalarial effect was exhibited at the highest dose tested. The decline in parasitemia, protecting PCV reduction, BW recovery, and prolonged MST in the treated mice justifies the folkloric use of GIE in the malaria treatment. Consequently, the GIE could be used as potential source to develop an alternative antimalarial drug.

## Data Availability

The data associated with this article are available at https://figshare.com/s/23bedd601f032193f059, DOI: 10.6084/m9.figshare.14316209.
